# Insecticidal Activities of Plants Extract Against Malaria Vectors in Hadiya Zone, Ethiopia

**DOI:** 10.1155/japr/9980264

**Published:** 2025-08-28

**Authors:** Anmut Assemie, Dasash Mulu, Alemayehu Getahun, Negalign Awoke, Workineh Muluken, Tigist Enyew, Amha Gebremariam, Yihenew Aynalem

**Affiliations:** ^1^Department of Biology, Wachemo University, Hossana, Ethiopia; ^2^Department of Statistics, Wachemo University, Hossana, Ethiopia; ^3^Department of Pediatrics and Child Health Nursing, Wachemo University, Hossana, Ethiopia

**Keywords:** *Allium sativum*, *Azadirachta indica*, mortality, vectors

## Abstract

Environmental changes due to global warming and human activities have negatively impacted malaria vector control in Hadiya zone, Ethiopia. Plants contain anthraquinoes. Flavonoids, glycosides, phenol, saponin, steroids, tannin, and terpenes that are target specific, rapidly biodegradable, ecofriendly, and less toxic to human health. The objective of the study was to evaluate the insecticidal activities of *Azadirachta indica* (neem) and *Allium sativum* L. (garlic) ethanol extracts against malaria vectors in the study area. Then, 20 g from each (*A. indica* and *A. sativum* L.) were extracted separately by ethanol solvents. The phytochemical analysis was evaluated from the crude sample based on standard methods. Then, insecticidal activities were evaluated by introducing the fourth instar larva at 50, 100, 150, 200, and 250 ppm concentrations, and data were subjected to probit analysis to determine the LC_50_ and chi-square test to check the significance of the mortality by R statistical software. The presence of phytochemical tests such as alkaloids, saponin, tannin, phenol, anthraquinoes, flavonoids, glycosides, steroids, terpenes, and flavonoids was obtained. The mortality of malaria vectors due to ethanol extract of *A. indica* and *A. sativum* was observed. The highest (90.66%) mortality was observed in the ethanol extract of *A. sativum* at 250 ppm concentration. *A. sativum* extracts have a significant effect only on the mortality of *Anopheles gambiae s.l* (*X*^2^ = 13.6, *p* = 0.008687) and *Anopheles pharoensis* (*X*^2^ = 11.002, *p* = 0.02655), but *A. indica* have a significant effect only on the mortality of An.*pharoensis* (*X*^2^ = 14.26, *p* = 0.00651). The lowest LC_50_ (39 ppm) was observed in the ethanol extract of *A. sativum*. So, *A. sativum* extract was highly toxic than *A. indica* extract and more effective in the reduction of malaria vectors but further studies will be conducted to determine the insecticidal activities at pupa and adult stages.

## 1. Introduction

Malaria vectors (*Anopheles* mosquitoes) are the main vectors that transmitted malaria causing protozoans such as *Plasmodium falciparum*, *Plasmodium vivax*, *Plasmodium ovale*, *Plasmodium malariae*, and *Plasmodium knowlesi* [1–4]. In the world, 212 million cases of malaria and 429,000 deaths were recorded in 2015. More than 80% of malaria cases occur in sub-Saharan Africa. In Ethiopia, about 68% of the total population lives in areas at risk of malaria [5]. In the Southern region of Ethiopia including Hadiya zone, the pooled prevalence of malaria was 19.19%; this was due to malaria vectors [6].

Plant extracts are sources of mosquito control agents due to the presence of bioactive compounds such as tannins, isoflavonoids, terpenes, steroids, and saponins [7, 8]. Bioactive compounds obtained from plant sources act as larvicides and play an important role in the interruption of the transmission of mosquito-borne diseases to the individual [9].

Plant parts are used directly through extraction, and essential oils may be extracted to repel mosquitoes [10]. Extracts of different plant are used for killing the larva of malaria vectors. The plants that have insecticidal activities on mosquitoes were *Citrullus colocynthis*, *Ocimum basilicum*, *Cymbopogon winterianus* Jowitt, *Dysoxylum malabaricum*, *Khaya senegalensis*, *Ficus benghalensis*, *Mentha piperita*, *Allium sativum*, and *Zingiber officinale*, and so forth [11, 12]. Bioactive compounds induce developmental changes and alterations to the midgut epithelium in mosquito vectors [13, 14].

The different challenges related to the efficacy of synthetic chemicals, resistance in the mosquito, and their risk on nontarget organisms have emphasized the generation of new strategies for vector-borne diseases [1, 2, 15, 16].

Secondary metabolites derived from plant extract are used as insecticidal activities, insect growth regulators, repellents, and oviposition attractants for the prevention and control of malaria disease transmission from patient to healthy, community to community, and country to country because of their low cost, risk-free feature, and easy to manage feature [9, 17].

Insecticidal natural products of plant origin have been produced in the past to control a variety of diseases and different plant materials are commonly consumed and have a long history of being utilized as traditional medicine. Among those, *Azadirachta indica* (neem) and *A. sativum* are of those plants that were seriously investigated over several years and used for centuries to fight infectious diseases and have antifungal, antiviral, antibacterial, antihelmantic, antiseptic, and anti-inflammatory properties [18–20]. The ethnobotanical knowledge regarding the use of the plant to handle mosquitoes and treat mosquito-related diseases, especially around our study area, is not well established. Therefore, the current studies used to determine the insecticidal activities *of A. indica,* and *A. sativum* against the larvae of malaria vectors.

## 2. Materials and Methods

### 2.1. Study Area

Plant part collection, the extraction process, and insecticidal activities were done at the Biology and Chemistry Lab Class of Wachemo University, Hadiya Zone, Ethiopia.

### 2.2. Larvae Collection

Larvae collection was conducted from different breeding sites of Hadiya Zone, Ethiopia. The study site was selected purposively based on altitude and temperature which are located between 1250–2500 m above sea level, and temperature between 21°C and 29°C. The collection of larval was carried out using a standard dipper (350) by lowered at 45° until one side was just below the water. Then, by using the water from the natural habitat, larvae were poured into plastic trays. The collected larvae were transferred to mosquito cages for identification at the field level. Then, we identify morphological by using the keys of Gillis and Coetzee [21].

### 2.3. Plant Collection and Extraction Process

The leaves of *A. indica* (neem) which were identified by experienced botanists were collected from any place which was present. The leaves of *A. indica (neem)* were washed with tap water, dried for 10 days and then change to powdered form. Then, 20 g of *A. indica* powder was mixed with 200 mL of ethanol and shaken using a shaking machine for 48 h at 30°C; then, the sample solutions were collected. The solution was concentrated using a rotary vacuum evaporator machine.

*A. sativum* (garlic) was collected and washed with tap water and then pilled, and it is slashed into smaller sizes to ease the drying process. The slashed *A. sativum* was spread on a newspaper and placed in the open cabinet for 20 days at 40°C in biology lab class. A weighing 20 g of the powdery form was extracted with 200-mL ethanol and covered with foil paper. After 2 weeks, the extract evaporated to remove the solvent. The extract was then stored in labeled specimen bottles for insecticidal assay [19].

### 2.4. Phytochemical Analysis

The presence of bioactive compounds that have a toxic effect on insects was screened using standard methods [22]. The reagent required for the screening of bioactive compounds were methanol, glacial acetic acid, ethanol, Mayer's reagent, hydrochloric acid, ferric chloride, sulfuric acid, ammonia, chloroform, distilled water, and iodine [22].

### 2.5. Bioassay Study

Five samples of each plant 5, 10, 15, 20, and 25 mg of the filtrates were prepared. The prepared sample was mixed with 100 mL of distilled water separately. For each plant extract, one beaker does not contain any treatment diluted and used as the control. Finally, 5, 10, 15, 20, and 25 mg of each sample were mixed with 100 mL of distilled water and mix them with one beaker which contains 100 mL of tap water. The final concentration was 50, 100, 150, 200, and 250 ppm.

Then, 10 fourth-stage larvae of malaria vectors were introduced into a glass beaker of 250-mL capacity containing different concentrations of test solutions. The treatments were observed after 6 h. If the larvae do not show any sign of movement after treatments, they were considered dead [23].

### 2.6. Statistical Analysis

After the bioassay study, all the observations become summarized and prepared for the inferential test, and mortality is calculated as follows:

 $$\begin{array}{c} \text{Percentage of mortality}=\frac{\text{number of dead larvae }}{\text{ number of larvae introduces}}\times 100. \end{array}$$

The average larval mortality data were subjected to probit analysis for calculating the toxicity parameters (LC_50_) and Chi-square using R software to determine their insecticidal efficacies. Mean mortalities of insect larva treated with crude extract of the plants were determined by analysis of variance using the R Version 4.3.1 (2023-06-16). *p* value less than was considered to be statistically significant.

## 3. Results

### 3.1. Phytochemical Screening

The analysis of *A. sativum*, and *A. indica* ethanol extracts shows positive results for nine and seven phytochemical tests, respectively. The qualitative phytochemical screening (presence or absence) of different phytochemical compounds in *A. sativum* and *A. indica* ethanol extracts were indicated in Table 1.

### 3.2. Insecticidal Activities

The mortality observed in the ethanol extract of *A. sativum* was range from 20.05% to 90.66% and *A. indica* 0 to 80.4% as shown in Table 2. The maximum mortality of ethanol extract was recorded in the *A. sativum* at a concentration of 250 ppm. In contrast to ethanol extract, no larva mortality in the negative control but the positive control exhibited 100% larva mortality. The mortality of malaria vectors due to the action of *A. indica* increased with an increase in concentration but this is not in the ethanol extract of *A. sativum.* The test of ethanol extract of *A. sativum* has a significant effect on mortality of *Anopheles gambiae s.l* (*X*^2^ = 13.6, *p* = 0.008687), and *Anopheles pharoensis* (*X*^2^ = 11.002, *p* = 0.02655), but not in *Anopheles funestus* (*X*^2^ = 3.9627, *p* = 0.4111). The test of *A. indica* has a significant effect only on *An. pharoensis* (*X*^2^ = 14.26, *p* = 0.00651).

The ethanol extracts of *A. sativum* have LC_50_ = 133.7 ppm, LC_90_ = 40.57 ppm, LC_95_ = 27.05 ppm, and LC_99_ = 11.04 ppm values against *An. gambiae s.l*, whereas *A. indica* have LC_50_ = 74 ppm, LC_90_ = 13.74 ppm, LC_95_ = 7.75 ppm, and LC_99_ = 2.18 ppm. The toxicity of *A. sativum* and *A. indica* against *An. pharoensis* shows (LC_50_ = 102.5, LC_90_ = 22.98, LC_95_ = 13.82, and LC_99_ = 4.5 ppm); (LC_50_ = 188, LC_90_ = 89.58, LC_95_ = 69.61, and LC_99_ = 39.87), respectively, as shown in Table 3.

## 4. Discussion

The first three stages (immature stage) of any mosquito lives in aquatic environments, which makes them attractive targets for insecticides during the prevention and control of mosquito-borne diseases. They are easier to control when they occur as juveniles in concentrated and contained habitats as compared to when they are in a widely dispersed adult population [24]. The identified malaria vectors in the present study were *An. gambiae s.l*, *An. pharoensis*, and *Anopheles funestus* which group under malaria vector in the study area.

The occurrence of malaria vectors in the study area was low as compared to the occurrence of malaria vectors in other countries/study areas [25–29]. This variation may be due to study period/season, way of larvae collection, time of larvae collection, way of identification, breeding sites, stage of identification, and biotic and abiotic factors.

The reduction of malaria vectors is important to reduce malaria diseases and many other vector-related diseases in sub-Saharan Africa including Ethiopia [30, 31]. Previously, an unlimited number of researchers do research to evaluate the toxicity of plant extract against malaria vectors in the world and get a positive effect on the control and prevention of malaria diseases [30].

The extracts of different plant materials have been studied for use as eco-friendly insecticides instead of eco-enemy synthetic insecticides [17, 32–38].

Research on the possible use of plant extract with different modes of action could be an alternative to prevent and control malaria disease and to reduce the risk of synthetic insecticides. *A. sativum* and *A. indica* are found in the major area of Ethiopia and are used as pest control. *A. sativum* and *A. indica* are now extensively used in medical aspects all over the world as insecticides including Ethiopia [19, 39, 40]. The study confirmed that the *A. sativum* and *A. indica* extracts have an effect on the larvae of malaria vectors according to WHO standards for testing potential larvicide effectiveness.

Phytochemical screening of the ethanol extract of *A. sativum* and *A. indica* shows the presence of alkaloids, saponin, tannin, phenol, anthraquinoes, flavonoids, glycosides, steroids, terpenes, and flavonoid. The presence of those secondary bioactive compounds in *A. sativum* and *A. indica* is active in the mortality of *An. gambiae s.l*, *An. pharoensis*, and *An. funestus*. Plants that contain secondary metabolites have biological activities [19, 40–42].

In the current study, 90.66% mortality was observed at 250 ppm due to the ethanol extraction of *A. sativum*, which is related to *Z. officinale* caused 100% mortality at concentrations of 125 ppm and below against *Culex pipiens* during the period 12–24 h posttreatment and LC_50_ 286, 76, 20, and 17 ppm after 3, 6, 9, and 12 h, respectively [43]. The essential oil of *Z. officinale* showed an effect against different mosquitoes with an LC_50_ range of 50.78–197 ppm [44], rosemary oil exhibited the highest toxicity on camel tick *Hyalomma dromedarii* (LC_50_: 12.3%), but neem and Cyperus oils revealed approximately the same toxicity recording an LC_50_ of around 29% on the second day [45] and fennel and green tea oils and their nanostructured lipid carriers have mortality against *Cx. pipiens* in the laboratory field conditions [46]. This variation may be due to exposure time, type of solvents, solvent-to-solute ratio, away of extraction, drying time, and test organisms.

In the current study, the mortality of *An. gambiae s.l* by ethanol extract of *A. sativum* and *A. indica* was high, which was related to N-hexane and chloroform extract of *Annona senegalensis* against *An. gambiae* and *Culex. quinquefasciatus* at 2500, 1250, 625, and 312.5 ppm concentration [47]; aqueous extract of *Moringa oleifera* against *An. gambiae s.s.* larvae at 2000 ppm concentration [48]; and aqueous and ethanol leaf extracts of *Hyptis suaveolens* against egg and larvae of *An. gambiae* at 1000, 500, 100, 50, and 5 *μ*g/mL concentration [49], 10% of *A. sativum* oils against *Cx. pipiens* [50], and *A. sativum* and *Z. officinale* oils against *Cx. pipiens* at 125, 250, 500, 1000, and 2000 ppm concentration [51].

The ethanol extract of *A. sativum* and *A. indica* was effective against the larvae of the identified malaria vectors similar to the leaf extract of *Typhonium trilobatum* [52]; leaf, flower, and whole plant extracts of *Jatropha curcas*, *Hyptis suaveolens*, *Abutilon indicum*, and *Leucas aspera* [53]; the leaf extract of *Mesua ferra* [54]; fruit extract of *Croton caudatus*; flower extract of *Tiliacora acuminate* [55]; flower extract of *Tagetes erecta* [56]; *Azadirachta indica* oil formulation [57, 58]; the leaf extract of *Millingtonia hortensis* [59]; leaf extract of *Tagetus minuta*, *Ageratum conyzoides*, and *Jatropha curcas* [60]; leaf extract of *Cucurbitacious* plant [61]; and leaf extracts of *Rhizophora mucronata* against *Anopheles stephensi*, *Cx. quinquefasciatus*, and *Aedes aegypti* mosquito vectors [62].

In the present study, ethanol extract of *A. sativum* and *A. indica* are effective against *An. gambiae s.l*, *An. pharoensis*, and *An. funestus*, which was also related to the study conducted by others [38, 42, 59, 63]. The plants used have no risk to environments and nontarget organisms, but the insecticide effect depends on the type of plants, methods of extraction, amount/dose, the solvents used, and exposure time.

## 5. Conclusion

In general, the ethanol extract of *A. sativum* was the best alternative for reducing the population of malaria vectors as compared to *A. indica* at the concentration of 50, 100, 150, 200, and 250 ppm. So using those plant extracts is the best alternative to reduce the risk of the synthetic chemical on the environment, in addition to reducing the population of malaria vectors. But further studies will be done on the insecticidal activities of ethanol and other solvent extract of *A. sativum* and *A. indica* against various stages (pupa and adult) of malaria vectors and other vectors under laboratory and field conditions by considering the physicochemical characterization of the breeding sites.

## Figures and Tables

**Table 1:** Phytochemical screening of ethanol crud extract.

**Secondary metabolites**	**Selected plant**	
	***Allium sativum***	***Azadirachta indica***
Alkaloids	**+**	**+**
Saponin	**+**	**+**
Phlobatannin	**−**	**−**
Tannin	**+**	**+**
Phenol	**+**	**+**
Anthraquinoes	**+**	**−**
Flavonoid	**+**	**+**
Glycosides,	**+**	**+**
Steroids	**+**	**+**
Terpenes	**+**	**−**
Cardenolides	**−**	**−**

*Note:* “+”means present and “–” means absent.

**Table 2:** Insecticidal activity of ethanol extracts of selected plant against malaria vectors.

**Malaria vectors**	**Con (ppm)**	**Mean mortality ± SE**	
		***Allium sativum***	***Azadirachta indica***
*Anopheles gambiae s.l*	50	2.05 ± 1.566	4.40 ± 0.887
	100	4.67 ± 0.890	5.20 ± 0.963
	150	3.02 ± 0.886	7.80 ± 0.263
	200	7.23 ± 0.556	8.00 ± 0.323
	250	9.58 ± 0.365	8.40 ± 0.223

Statistical analysis	*X* ^2^	13.6	6.7736
	*p* value	0.008687	0.1483

*Anopheles pharoensis*	50	4.33 ± 0.667	0.00 ± 0.00
	100	3.80 ± 0.950	1.40 ± 2.56
	150	5.65 ± 0.465	3.33 ± 0.984
	200	8.12 ± 0.557	6.20 ± 0.156
	250	9.68 ± 0.337	6.00 ± 0.066

Statistical analysis	*X* ^2^	11.002	14.26
	*p* value	0.02655	0.00651

*Anopheles funestus*	50	6.62 ± 0.333	3.20 ± 1.025
	100	7.33 ± 0.482	5.00 ± 0.785
	150	8.0.66 ± 0.556	4.02 ± 0.895
	200	9.33 ± 0.332	7.40 ± 0.433
	250	9.66 ± 0.566	8 0.40 ± 0.225

Statistical analysis	*X* ^2^	3.9627	6.2648
	*p* value	0.4111	0.1802

Abbreviations: *p* value, probability value; ppm, parts per million; *X*^2^, chi-squared value.

**Table 3:** Lethal concentration of crude extracts of the plants.

**Malaria vectors**	**Plants extracted**	**Toxicity in ppm**			
		**LC**_**50**_	**LC**_**90**_	**LC**_**95**_	**LC**_**99**_
*Anopheles gambiae s.l*	*Allium sativum*	133.7	40.57	27.05	11.04
	*Azadirachta indica*	74	13.74	7.75	2.18
*Anopheles pharoensis*	*Allium sativum*	102.5	22.98	13.82	4.5
	*Azadirachta indica*	188	89.58	69.61	39.87
*Anopheles funestus*	*Allium sativum*	39	16.23	5.66	1.89
	*Azadirachta indica*	96	63.55	26.88	10.26

Abbreviations: LC_50_, lethal concentration that kills 50% of the exposed larvae; LC_90_, lethal concentration that kills 90% of the exposed larvae; LC_95_, lethal concentration that kills 95% of the exposed larvae; LC_99_, lethal concentration that kills 99% of the exposed larvae.

## Data Availability

All the data used to support the findings of this research were included in the manuscript.
